# The language of isolation: a commentary on Westram et al., 2022

**DOI:** 10.1111/jeb.14029

**Published:** 2022-09-05

**Authors:** Roger K. Butlin

**Affiliations:** ^1^ School of Biosciences The University of Sheffield Sheffield UK; ^2^ Department of Marine Sciences, Tjärnö Marine Laboratory University of Gothenburg Strömstad Sweden

Good communication needs a common language. As Harrison (2012) pointed out, the ‘language of speciation’ has a complex evolutionary history, sometimes resulting in both modification and diversification in the use of terms. There is a risk that this erodes the effective exchange of ideas. It is surprising that Harrison did not discuss the meaning of ‘reproductive isolation’. He seems to have assumed, along with many others, that there is a common understanding of this term. In fact, it is not an easy term to define and the conversation started here by Westram et al. (2022) is both valuable and timely.

Multiple terms are in use to describe the extent to which populations are connected by interbreeding and gene flow: ‘reproductive isolation’ itself, various terms for ‘components of reproductive isolation’ in a temporal sequence derived from Dobzhansky's seminal table (Dobzhansky, 1937, pp. 231–232; prezygotic isolation, postzygotic isolation and a series of finer divisions), a partly independent categorization into ‘intrinsic’ vs ‘extrinsic’ components and a set of terms related to ‘barriers to gene flow’, such as ‘barrier traits’, ‘barrier loci’ and ‘barrier effects’. Having multiple terms is potentially confusing, but it can also be very helpful: if these terms have clear and distinct meanings, they can be used to make descriptions more precise and so to communicate more reliably. They can also guide decisions about what to measure empirically and help to show how different types of empirical information fit together. Westram et al. (2022) define ‘reproductive isolation’ as a quantitative measure of the effect that genetic differences between populations have on gene flow, specifically aiming to bring together what they describe as the ‘organismal focus’ and the ‘genetic focus’ revealed in their survey of practitioners. Here, I argue that better communication can be achieved by using the available terminology to describe different aspects of the evolutionary independence of populations rather than finding a single definition of reproductive isolation that incorporates all aspects.

The issue of interest is the evolutionary independence of populations. This depends on gene flow, which tends to homogenize neutral variation, restrict responses to divergent selection and facilitate shared responses to common selection pressures. Populations of the same species have high levels of interdependence, whereas populations of different species can evolve independently (in a genetic sense: their evolutionary trajectories may well be constrained by ecological factors, but that is not the point at issue here). At this conceptual level, there is broad agreement and ‘reproductive isolation’ is used in a general sense to describe a continuum of relationships (Stankowski & Ravinet, 2021). The problems arise when trying to be more precise, especially when looking for quantitative comparisons.

One key distinction to consider lies in the entities for which a quantitative measure is required. Reproductive isolation is generally considered a property of a pair of populations, but gene flow is only meaningful at the level of genes. In a theoretical construct of two populations connected by gene flow but experiencing no selection, it might be true that all loci have the same expected gene flow. However, such pairs of populations do not exist in nature. Habitat variation is ubiquitous, leading to divergent selection on a subset of loci and so lower gene flow for these loci. Even in the absence of divergent selection, gene flow would be expected to vary due to beneficial and deleterious mutations appearing independently in the two populations. Gene flow at neutral loci is influenced by linkage to these selected variants, and so there is no single value that applies to all neutral loci.

A second issue is that populations are, in the great majority of cases, separated in space. Often, they are also separated by physical barriers to dispersal. Both space and physical barriers reduce gene flow and create some degree of evolutionary independence between populations. In his original classification of ‘isolation’, Dobzhansky (1937) began with a distinction between ‘geographical’ and ‘physiological’ (= ‘reproductive’) isolation, arguably considering them to be two parts of one whole. Mayr (1963, p. 91), in contrast, was adamant that they should be kept separate. Part of the reason for this desire for a clear separation seems to be that Mayr saw reproductive isolation as a set of ‘protective devices' (Mayr, 1959). Dobzhansky (1937) coined the term ‘isolating mechanism’, also tending to emphasize the idea that traits evolved to protect what he and Mayr saw as integrated gene pools. This idea was challenged, especially by Paterson (1978) and Littlejohn (1989), on the basis that many traits that contribute to a reduction in gene flow between populations evolved for reasons unrelated to this outcome. It is instructive (perhaps rather depressing) to note that general acceptance of this argument has not resulted in the suppression of the misleading terminology of isolating mechanisms: more than 40 years on, this remains the standard terminology in introductory evolutionary biology texts.

The distinction between geographical isolation (including both distance and physical barriers) and reproductive isolation (‘owing to genotypically conditioned differences between … populations’, Dobzhansky, 1951) is actually not clear‐cut. Wiens (2004) made this point strongly. He used the example of populations adapted to a specific altitudinal zone: a valley presents a physical barrier to gene flow only if the valley bottom is below the lower range limit of the species, and a ridge is only a barrier if it is above the range limit. What constitutes a physical barrier clearly depends on the biological characteristics of the populations in question. It is hard to draw a clear line between this type of barrier and the many ‘genotypically conditioned’ barriers created incidentally by divergence between populations for reasons unrelated to the prevention of gene flow.

These two points together undermine the idea that reproductive isolation can be measured as a reduction in gene flow relative to some reference, *m*, as implied by the definition in Westram et al. (2022) and the core formula they use (their Equation 1):

(1) $${\mathrm{RI}}_{2d}=1-\frac{m_{e}}{m}$$

Here, *m* reflects the physical opportunity for gene flow, encapsulating the geographic component of isolation as it influences neutral alleles, with other components of isolation reducing gene flow further to an ‘effective’ level, *m*_*e*_ (for neutral loci unlinked to directly selected loci). But, geographic isolation is not conceptually distinct from reproductive isolation: in the scenario described by Wiens (2004), *m* between two populations on either side of a valley is <0.5 partly due to distance and partly because the adaptations of these populations reduce their ability to pass through the valley floor. If neutral alleles are rarely free from the influence of linked selected loci, then *m* is conceptually difficult and also probably cannot be estimated empirically. Westram et al. (2022) recognize this empirical difficulty. They also argue that *m*_*e*_ can be estimated in many situations, not for populations but for loci within them, and that it is a meaningful quantity in itself, even if neutral loci that are not linked to any selected loci are rare in the genome. This usage of effective gene flow is fully compatible with the approach that I advocate below, but I argue that it can usefully be extended to loci under selection.

Gene flow between two populations requires that individuals in reproductive condition meet, mate and produce viable and fertile offspring. Up to the point of F1 fertility, anything that interferes with these steps has an equal effect on all loci in the genome (with the possible exception of loci with sex‐specific transmission, see below). After recombination in the F1, fitness and mating patterns in subsequent generations also have impacts on gene flow but they do so in a locus‐specific manner. This is true not only for genes that directly influence fitness but also for neutral loci because each neutral locus has a distinct set of linkage relationships to selected loci. This provides a conceptual separation between effects that operate before recombination, which can properly be considered as quantifiable attributes of a pair of populations, and measures of gene flow, which are attributes of the genes within a pair of populations. This is also a pragmatic separation because the measures that Westram et al. (2022) consider part of the ‘organismal’ view of reproductive isolation are typically measured for samples of individuals from populations, up to F1 fitness, whereas gene flow, in their terms the ‘genetic’ measure of reproductive isolation, is measured for individual loci or sets of loci (and often on longer time‐scales). Rather than conflating these two things, and worrying that it is hard to make the different types of measurement equivalent, it is surely more constructive to keep them separate, measure them separately and try to understand their relationship. The separation also helps in relation to the different types of question that are addressed in speciation research. For example, Coyne and Orr (1989, 1997) asked about the rates of evolution of assortative mating and hybrid inviability or sterility, which are measures of the success of interbreeding, whereas a question about the impact of hybrid sterility on gene exchange in different parts of the genome requires measures of realized gene flow, such as might be obtained in hybrid zone analyses (e.g. Janousek et al., 2012).

With these considerations in mind, it is possible to use the available terminology to describe different aspects of the overall phenomenon of interest, that is the varying degrees of evolutionary independence between pairs of populations (Table 1). The definitions here build on those used by Butlin and Smadja (2018) who tried to be clear about the terminology of ‘barriers to gene flow’ but who did not tackle the meaning of ‘reproductive isolation’. ‘Reproductive isolation’ can still be used, as it is now, in a general conceptual sense, alongside a more precise and quantitative definition (of the type proposed by Westram et al., 2022 or in Table 1). This sort of dual use of terms is actually widespread and familiar (Brigandt, 2020). It is how we use terms like ‘species’ and ‘fitness’, introducing more constrained definitions when needed.

**TABLE 1:** Terminology of isolation and gene flow

Term	Definition	Comment
Isolation	Reduction in the production of viable and fertile offspring between, relative to within populations	$I=1-\frac{O_{b}}{O_{w}}$ *O*_*b*_ is the probability of producing a viable and fertile between‐population hybrid offspring. *O*_*w*_ is the probability of producing a viable and fertile offspring with a population
Component of isolation	A contribution to overall isolation arising from a step in the reproductive process, including the meeting of individuals	A typical (not exhaustive) hierarchy of components: Geographical isolation Spatial isolation Physical barrier Reproductive isolation Prezygotic isolation Ecological isolation Habitat association Allochronic isolation Mating pattern Post‐mating, prezygotic isolation Postzygotic isolation F1 inviability F1 sterility The strength of each component and the combination of components to give overall isolation can be estimated following Sobel and Chen (2014), for example.
Barrier to gene flow	Anything that causes a reduction in gene flow at a locus, between two populations	$B=2\left(0.5-m_{e}\right)$ 0.5 is the expectation under random mixing and the factor of 2 is introduced so that the barrier strength varies from 0 (no barrier) to 1 (complete barrier)
Barrier trait	A trait that contributes to a component of isolation and/or a barrier to gene flow	Typically, a component of isolation is an effect (sensu Williams 1966) of the evolution of one or more barrier traits but selection can also act on barrier traits to enhance or combine barrier effects (Butlin & Smadja, 2018)
Barrier locus	A locus that contributes to a barrier trait or otherwise has a direct impact on gene flow	A barrier locus may cause a reduction in gene flow itself or in combination with other loci
Barrier effect	The reduction in gene flow caused by a barrier trait or locus	Typically, a barrier effect is genomically localized, at and close to the barrier loci involved

The definitions proposed in Table 1 do not remove the many practical difficulties that Westram et al. (2022) identify for both ‘organismal’ and ‘genetic’ estimates. The main effect of these definitions is to formalize the separation of viewpoints that was shown to be widespread in the speciation research community by the survey reported in Westram et al. (2022). The organismal view, here reproductive isolation, is also compatible with the idea of ‘interbreeding’ in the standard definition of the biological species concept. Another significant change in viewpoint is that reproductive isolation becomes a component of isolation, reviving Dobzhansky's (1937) original formulation. It is widely recognized that components of isolation are difficult to separate cleanly and this certainly applies to the geographical‐reproductive separation, as noted above. Sobel et al. (2010) and Sobel & Chen (2014)) have discussed this issue previously, arguing for a distinction between ‘effective geographical isolation’, which is not dependent on the intrinsic characteristics of the species, and ‘ecogeographic isolation’, which is. However, it is hard to imagine any form of geographical isolation that is not dependent on attributes of the populations considered: the effect of spatial separation depends on dispersal and the effect of a physical barrier depends on adaptation, as in the altitudinal range example used by Wiens (2004). Westram et al. (2022) show how physical and genetic barriers to gene flow can be hard to distinguish and can be measured in the same units. Therefore, I argue that the inclusion of geographic isolation as a component of overall isolation, alongside reproductive isolation, is more coherent than their separation into distinct conceptual bins.

Using the language of isolation, on the one hand, and barriers to gene flow, on the other, to refer to distinct aspects of the relationships between populations requires a shift in thinking. Despite noting the organismal—genotypic divide, Westram et al. (2022) follow a common trend in the speciation research literature that views measures of reproductive isolation as direct reflections of gene flow. This is explicit in Sobel and Chen's influential paper (2014). They use the term ‘reproductive barrier’ or ‘reproductive isolating barrier’ in order to avoid using ‘isolating mechanism’. These terms are equivalent to ‘component of reproductive isolation’ in Table 1. Quoting Coyne and Orr (2004), they say, ‘The purpose of calculating the strength of a reproductive isolating barrier is to estimate how much gene flow is reduced by a barrier’ (Sobel & Chen, 2014, p.1512, although Westram et al., 2022 argue that their measures do not achieve this aim). However, they also note (p.1511) that, ‘Early work on the nature of reproductive barriers sought to quantify the degree to which crossing barriers prevented hybridization or resulted in hybrid unfitness’ and that this is the way reproductive isolation is used in the classic comparative analyses of Drosophila speciation by Coyne and Orr (1989, 1997) and see Matute & Cooper (2021). In a sense, I am advocating a return to this earlier usage for reproductive isolation, while reserving the more recent barrier terminology to describe gene flow.

When isolation is complete, *I* = 1, there will be no gene flow at any locus, *m*_*e*_ = 0 throughout the genome. The other extreme (*I* = 0, *m*_*e*_ = 0.5) is less interesting because it probably never applies to two populations in nature that are spatially or temporally separated, or phenotypically distinguishable. Between these extremes, effective gene flow must decline, on average, as isolation increases but with much variation among loci. The nature of this variation depends on many interesting factors, including the components of isolation and the barrier traits and loci involved. These factors will also influence the shape of the relationship between isolation and average effective gene flow. Measuring components of isolation, determining the barrier traits responsible and their genetic basis and measuring their barrier effects are all needed for a full understanding of speciation. The key goal (Westram et al., 2022) of comparability across systems is more likely to be achieved with this separation of elements. Part of the concern in Westram et al. (2022) is about having one term (reproductive isolation) with multiple meanings, but this can be resolved by distinguishing the terminology of isolation from the terminology or barriers to gene flow.

If a barrier to gene flow causes a reduction, this must be relative to some reference level. In Table 1, I suggest using free gene exchange (0.5) as this reference. This is also a departure from the standard reference (*m*) used by Westram et al. (2022) and others, but it is the reference used by Sobel and Chen (2014, p.1514: ‘Perhaps the most important advantage of using RI_4_ is that isolation values obtained are intuitive because they represent the proportional reduction in gene flow relative to expectations under random mating’). It has two advantages, it emphasizes that geographical isolation is a part of overall isolation whose barrier effect needs to be measured, as with any other component, and it avoids reliance on a reference quantity, *m*, for which the meaning is unclear and the value hard to estimate. If *m* can be estimated for a locus or set of loci, then 0.5‐*m* becomes a measure of the barrier effect due to the geographic component of isolation. Viewed in this way, the language of barriers is applicable to barrier loci themselves, rather than being restricted to neutral loci as proposed by Westram et al. (2022).

A clear consequence of this way of thinking is that some isolation exists between all pairs of populations, including populations of the same species. This certainly reflects reality: conspecific populations are typically isolated by some combination of space, physical barriers and genetic differentiation. The conceptual model in which speciation begins with one fully mixed population and proceeds to two such populations that are completely reproductively isolated does not reflect this reality. Instead, the starting point is a network of populations with varying degrees of isolation and speciation consists of changes in the strengths of barriers to gene flow between pairs in the network, up to the point where a subset of populations become fully independent (see Harvey et al., 2019 and Huang, 2020 for related discussions). This way of thinking demonstrates that there is a need to describe both isolation and barriers to gene flow at the level of network subsets as well as at the level of pairs of populations. Indeed, this is what authors often mean when they discuss isolation or barriers ‘between species’ or ‘between incipient species’. The problem is related to the spatially continuous situations that Westram et al. (2022) discuss which, at least to some extent, can be broken down into networks of demes. The problem is different for isolation and for gene flow, because of the different time‐scales over which they are measured. The solution may lie in methods similar to those used in landscape genetics, which are actually designed to deal with the geographical component and some habitat‐related components of isolation within species (e.g. McRae, 2006, Fenderson et al., 2019). For practical estimation of *m*_*e*_, methods that work over long time‐scales effectively integrate over networks of populations, as argued by Westram et al. (2022). If barriers to gene flow are defined without reference to *m*, they provide direct measures of barrier strength. Whatever measure is used for isolation or barrier effects between these larger units, interpretation must take account of the non‐zero isolation (or barrier effect) that exists between populations within the units.

Both isolation and barriers to gene flow between a pair of populations may be asymmetrical, for example because the fitness of an individual (or allele) from population A is lower in habitat B than the fitness of a B individual (or allele) in habitat A. There may also be differences between directions of cross: A female × B male matings may be more frequent than B female × A male matings. This complexity is only briefly addressed by Westram et al. (2022). Asymmetries can alter the links between isolation and gene flow, providing another argument for keeping them separate. Sobel and Chen (2014) briefly discussed this issue (their Appendix C). Some parts of the genome might be influenced differently by these asymmetries. For example, the male F1 offspring of an A male × B female cross in a taxon with XY sex determination contains Y^A^, X^B^ and plastids from B, but if the cross is B male × A female, they contain Y^B^, X^A^ and A plastids. Any difference in components of isolation between the two reciprocal crosses potentially violates the expectation that breakdown of any step in interbreeding up to F1 fertility influences all parts of the genome equally. An average isolation between reciprocal crosses (following Sobel & Chen, 2014, Appendix C) may be meaningful, but will lose information on directionality for sex‐specific elements of the genome.

The analyses in Westram et al. (2022) are very helpful in thinking about barriers to gene flow and their measurement under various scenarios. However, they highlight the difficulties around equating reproductive isolation and a reduction in gene flow relative to a reference that is based on an expected level of neutral gene flow. Adjusting to think about isolation and gene flow separately, and including spatial separation and physical barriers as part of total isolation, does not resolve all of the issues by any means, but it can provide a way forward, both conceptually and empirically.

## CONFLICT OF INTEREST

The author declares no conflict of interest.

### PEER REVIEW

The peer review history for this article is available at https://publons.com/publon/10.1111/jeb.14029.

### DATA AVAILABILITY

No data are associated with this article.
